# FMLRC: Hybrid long read error correction using an FM-index

**DOI:** 10.1186/s12859-018-2051-3

**Published:** 2018-02-09

**Authors:** Jeremy R. Wang, James Holt, Leonard McMillan, Corbin D. Jones

**Affiliations:** 1 Department of Genetics, University of North Carolina at Chapel Hill, CB 3280, 3144 Genome Sciences Building, 250 Bell Tower Dr, Chapel Hill, 27599 NC USA; 2 Department of Computer Science, University of North Carolina at Chapel Hill, Chapel Hill, NC USA; 3 Department of Biology and Integrative Program for Biological and Genome Sciences, University of North Carolina at Chapel Hill, Chapel Hill, NC USA

**Keywords:** de novo assembly, Hybrid error correction, Long read, Pacbio, BWT, FM-Index

## Abstract

**Background:**

Long read sequencing is changing the landscape of genomic research, especially *de novo* assembly. Despite the high error rate inherent to long read technologies, increased read lengths dramatically improve the continuity and accuracy of genome assemblies. However, the cost and throughput of these technologies limits their application to complex genomes. One solution is to decrease the cost and time to assemble novel genomes by leveraging “hybrid” assemblies that use long reads for scaffolding and short reads for accuracy.

**Results:**

We describe a novel method leveraging a multi-string Burrows-Wheeler Transform with auxiliary FM-index to correct errors in long read sequences using a set of complementary short reads. We demonstrate that our method efficiently produces significantly more high quality corrected sequence than existing hybrid error-correction methods. We also show that our method produces more contiguous assemblies, in many cases, than existing state-of-the-art hybrid and long-read only *de novo* assembly methods.

**Conclusion:**

Our method accurately corrects long read sequence data using complementary short reads. We demonstrate higher total throughput of corrected long reads and a corresponding increase in contiguity of the resulting *de novo* assemblies. Improved throughput and computational efficiency than existing methods will help better economically utilize emerging long read sequencing technologies.

## Background

*De novo* genome assembly has benefitted dramatically from the introduction of so-called “long” read sequencing technologies. These technologies, such as SMRT sequencing by Pacific Biosciences (Pacbio) and nanopore sequencing platforms by Oxford Nanopore Technologies, produce reads typically 10s of kilobases instead of hundreds of bases. These reads can span repetitive or low-complexity regions of the genome previously unresolvable using only “short”-read next-generation sequencing. Unfortunately, the relatively high error rate of these long-read technologies introduces new informatics and analysis challenges. Effective and efficient methods are necessary to correct these errors in order to realize the potential of these long reads for whole genome assembly [1–4].

As the size of long read datasets and genomes undergoing *de novo* assembly increases, the performance of hybrid long read correction and assembly methods becomes increasingly important. For genomes of more complex eukaryotes and mammals, the computational resources required for effective *de novo* assembly are staggering and difficult to coordinate. This is driven largely by the pairwise overlap step required by all modern long read assemblers. The time required to overlap these long reads with one another increases quadratically relative to the number of reads. While novel methods such as MHAP [5] and Minimap [6] aim to improve this, in practice, the computational time and memory required are often prohibitively expensive.

Pre-assembly correction dramatically simplifies the subsequent overlap and layout of long reads for assembly by reducing the variance that must be accounted for in the overlapping step. In particular, long reads having undergone error correction are likely to share much longer identical stretches that can be used to efficiently find confidently overlapping reads. Fundamentally, the longer and more accurate these corrections are, the more quickly and accurately the long reads can be assembled.

Long read correction algorithms can be broadly classified as either self-correction or hybrid correction algorithms. Self-correction algorithms correct long reads using only other long read sequences. Self-correcting algorithms, including Sprai [7], LoRMA [8], HGAP [1], and PBcR [3] align the long reads to each other and generate a consensus sequence. In order to generate an accurate consensus, these methods require relatively high coverage of long read sequence to overcome the high error rate. Unfortunately, the relatively high cost per *accurate* nucleotide for long-read sequencing technologies means that deep sequencing using only long reads is expensive.

In contrast, hybrid correction algorithms use short-read sequencing of the same sample to complement and correct the long reads. Short-read sequencing has fewer sequencing errors, costs less per base sequenced, and thus the cost per *accurate* nucleotide is much lower. Many hybrid error correction methods act similar to scaffolders in that they require the assembly of complementary short read data first, then alignment between long reads and short-read unitigs or contigs. These approaches, while reasonably effective, suffer from two classes of problems. First, they incur the same type of disadvantages a short-read only assemblies in that low-complexity and repetitive elements larger than the size of the short reads cannot be reliably resolved. When short reads are preassembled, this bias can “correct” long read with incorrect sequence, confounding assembly. Second, short read assembly followed by pairwise alignment/overlap of long reads with short-read contigs is often significantly slower than direct long-read error correction.

Hybrid correction algorithms include LoRDEC [4], ECTools [9], Jabba [10], CoLoRMap [11], and Nanocorr [12]. Other methods, including Cerulean [13], DBG2OLC [14], and hybridSPAdes [15] perform hybrid assembly of long- and short-read data but do not explicitly correct errors in the long-read sequences. These hybrid methods are often able to construct more accurate and contiguous assemblies than exclusively long-read assembly methods at substantially lower cost. ECTools [9] and Nanocorr [12] are based on the same underlying methodology, but designed for Pacbio and nanopore sequences, respectively. They perform a full alignment between short and long reads, but are currently deprecated and take prohibitively long to run for anything larger than microbial genomes, so they were not considered further.

For error correction or assembly methods to be useful for large, complex genomes that are biomedically or economically important, the key challenge is performing as accurate an assembly as possible, as quickly as possible, and using as few computational resources as possible. Current methods often require prohibitively large computational resources. Given that finding the appropriate parameters for an assembly is often an iterative process, these high computational costs are a barrier.

## Methods

We introduce a new hybrid method for correcting errors in long-read sequences called FM-index Long Read Corrector (FMLRC). The main contribution of our method is the application of an FM-index built from a multi-string Burrows-Wheeler Transform (BWT) [16] of the short-read sequencing datasets. The FM-index enables arbitrary length *k*-mer searches through the dataset, allowing for FMLRC to retrieve *k*-mer frequencies from the short-read dataset in *O*(*k*) steps. Unlike other data structures, the length of *k* is not fixed during construction of the FM-index but is instead selected at run-time. As a result, FMLRC uses the FM-index to *implicitly* represent *all* de Bruijn graphs [17] of the short-read sequencing dataset. These de Bruijn graphs are then used to correct regions in the long reads that are not supported by the short-read sequencing dataset.

**Fig. 1:**
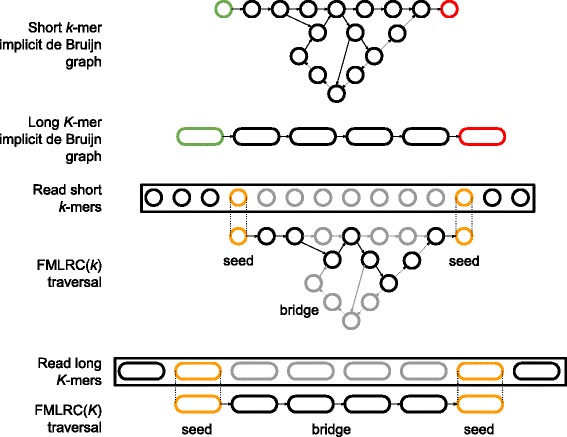
Illustration of the seed-and-bridge correction strategy using short and long *k*-mers. *Implicit* de Bruijn graphs with arbitrary *k* can be inferred from an FM-index. The use of a short, fixed *k* often does not resolve “hairball” and other structures in the graph caused by low-complexity and repetitive genomic elements. Longer *K*-mers may dramatically simplify the bridging step if sufficiently long seeds can be found. Illustrative seed-and-bridge paths are shown for short *k*-mer and long *K*-mer graphs. Seed *k*-mers are shown in orange, and the correct path in black. The two-pass (*k*, *K*) seed-and-bridge correction implemented in FMLRC allows the correction of short, nonrepetitive segments in the first pass, then seeding larger *K*-mers and bridging to resolve more complex sequences

Two secondary contributions arise as a result of the first. FMLRC uses the single FM-index data structure to perform two correction passes over each read: first with a short *k*-mer and second with a longer *K*-mer. Secondly, the specific parameters of the correction algorithm are dynamically adjusted to match the *k*-mer frequencies for a given read at run-time. FMLRC takes as input a BWT of the short-read sequencing dataset. It constructs a single FM-index in memory that is shared across all processes. Each process individually corrects one read at a time by applying common de Bruijn graph correction methods (namely seed-and-extend or seed-and-bridge) using the shared FM-index. These de Bruijn correction methods require both a *k*-mer size and frequency thresholds to determine whether a *k*-mer is present in the graph. FMLRC dynamically adjusts these thresholds at run-time for each pass over a long read. A single process will correct the read using the implicit short *k*-mer de Bruijn graph and then the implicit long *K*-mer de Bruijn graph before writing the corrected result to disk. An overview of this approach is shown in Fig. 1.

FMLRC is a publicly available C++ program^1^. The implementation requires construction of a BWT of the short-read dataset in the run-length encoded format of the msbwt package^2^.

### Advantages of the FM-index

FMLRC can be classified as a de Bruijn graph-based, hybrid read corrector, meaning it uses *k*-mer frequencies from a short-read sequencing dataset to correct errors in a long-read sequencing dataset. Generally speaking, most de Bruijn graph implementations are static and require a fixed *k*-mer size and pruning threshold to be defined during the construction of the de Bruijn graph [17].

The main advantage of FMLRC is that it uses an FM-index as the underlying de Bruijn graph implementation. FMLRC builds an FM-index [18] from a BWT [19] of a short-read sequencing dataset to correct a long-read sequencing dataset. These data structures have been previously used for short-read self-correction in FMRC [20], but it has not been previously applied to long-read error correction.

The FM-index is advantageous because many of the correction parameters are not properties of the data structure itself and can instead be defined and/or dynamically adjusted at run-time. First, FM-index queries are not fixed to a single *k*-mer size, allowing FMLRC to construct one FM-index and use it for all *k*-mer queries. Secondly, the FM-index is built from a BWT that is a lossless encoding of the original reads, meaning that no *k*-mers are “pruned” as they commonly are in a de Bruijn graph. This pruning is usually accomplished by removing all *k*-mers with a frequency less than a fixed threshold. Instead, FMLRC dynamically calculates thresholds for each long read and decides whether a *k*-mer is “pruned" at run-time. The combination of these two properties means the FM-index implicitly represents *all* de Bruijn graphs for the short-read sequencing dataset.

FMLRC creates an in-memory FM-index by scanning the BWT from disk. There are many different implementations of in-memory FM-indices that have varying trade-offs between memory usage and CPU time to perform a *k*-mer lookup. FMLRC currently has two FM-index implementations. The default FM-index implementation uses bit arrays and rank operations to enable fast *k*-mer lookups. This primary implementation sacrifices memory usage to increase computational performance. The second FM-index implementation is a traditional sampled FM-index that allows users to set the sampling rate, leading to longer computations with a smaller memory footprint. The two FM-index implementations produce identical corrected read results, and we present only the results from the primary implementation in our performance results.

For our results, we constructed the BWTs using a combination of *ropebwt2* [21] and the *msbwt* package^3^. While this particular format stores only the original read sequences, we must consider both the forward and reverse-complement sequences when performing *k*-mer queries. Every time we refer to a *k*-mer query, FMLRC is actually querying both the forward and reverse-complement sequences and adding their frequencies together prior to performing any checks.

### De Bruijn graph-based correction

FMLRC accesses implicit, pruned, *k*-mer de Bruijn graphs through the FM-index. While the de Bruijn graph-based correction of FMLRC is similar to that of LoRDEC [4], we briefly describe it here for completion and for reference in the following sections. Given a long read and a de Bruijn graph, the first step is to classify all *k*-mers in the long read as either *weak* or *solid*. In general, *solidk*-mers are supported by the de Bruijn graph and *weakk*-mers are not. For each *k*-mer in the long read, its *k*-mer frequency is retrieved from the de Bruijn graph. If that frequency is below a threshold, *t*, it is consider weak and otherwise it is considered solid. Weak regions are consecutive weak *k*-mers in the long read. Solid regions are consecutive solid *k*-mers in the long read.

Weak regions can be flanked by zero, one, or two solid regions. If a weak region has no flanking solid regions, the entire read is one large weak region with no solid *k*-mers to initialize a traversal of the de Bruijn graph. As a result, these reads are not changed because there are no start points for a de Bruijn graph traversal.

If a weak region has one flanking solid region, then it is either a head or tail weak region in the read. In either case, the solid *k*-mer closest to the weak region is used as a “seed” *k*-mer for traversing the de Bruijn graph. FMLRC performs a depth-first traversal of the de Bruijn graph from this seed using an expected path length based on the size of the weak region and returns any found paths (seed-and-extend). If a weak region has two flanking solid regions, FMLRC uses the two closest *k*-mers from each solid region as “seed” and “target” *k*-mers (seed-and-bridge). FMLRC then performs a depth-first traversal from the seed *k*-mer and returns any paths that connect to the target *k*-mer. If no path is found, FMLRC attempts to extend backwards from the target to the seed *k*-mer, which may resolve additional bridges that have excessive branching close to the seed *k*-mer. If any paths are returned from a de Bruijn graph traversal, the paths are compared to the original weak region and the one with the smallest edit distance is chosen to replace it. If no paths are returned, then no change is made to the long read at that region. In all de Bruijn graph traversals, we prevent exponential traversal time by enforcing a branching limit, *L*. Typically, the parameters *t* and *L* are either constant values in a program or user-defined static values.

### Differences in the short and long passes

One of the key differences in FMLRC compared to other approaches is that it accesses two different de Bruijn graphs though the FM-index and dynamically adjusts the parameters of the correction algorithm to adjust for differences in the graphs. FMLRC performs two passes: the first with a short *k*-mer size and the second with a longer *K*-mer size. For FMLRC, the two passes are programmatically identical with the value of *k* or *K* passed as a parameter. For brevity, we describe the differences in each pass using parameter *k* noting that replacing *k* with *K* describes the second pass of our method. Additionally, we describe any dynamic variables as functions of *k*, the implicit *k*-mer de Bruijn graph, and other user-defined constants.

In general, the short *k*-mer pass does the majority of the correction for FMLRC, whereas the longer *K*-mer pass tends to correct repetitive, low-complexity regions within the long read. To provide some intuition behind why the long pass improves the results, we focus on the differences in de Bruijn graphs representing the same data but with two different *k*-mer lengths. In general, two distinct paths will be merged in a *k*-mer de Bruijn graph if they share a pattern that is at least *k* long. This is because the nodes along that shared region will be identical. At the ends of the shared region, there will be two paths emerging representing the differences at the edge of the shared regions.

When the same sequences are viewed through a longer *K*-mer de Bruijn graph, the number of merged, ambiguous paths strictly decreases because an increasing amount of similarity is required for the paths to become merged in the graph. This effect is illustrated in Fig. 1. In practice, short *k*-mers are often long enough to uniquely identify most areas of the genome. However, genomic characteristics such as low-complexity sequence, gene families, or repeat regions are difficult to traverse using short *k*-mers. Thus, our method uses the larger *K*-mer to bridge weak regions composed of repeated or low-complexity sequences that are computationally expensive to fully traverse using a small *k*-mer.

In addition to changing the value of *k* in the two passes, other parameters are adjusted as well to match the different *k*-mer sizes. First, the threshold, *t*, determining whether a *k*-mer is weak or solid is dynamically adjusted for each long read. FMLRC uses a dynamic threshold based on the *k*-mer frequencies in the long read. First, there is an absolute minimum, user-defined *k*-mer frequency, *T*, that is required for any *k*-mer to be consider solid. Second, the frequency of any *k*-mer greater than this absolute minimum is added to a list and used to calculate a median solid frequency, *m*, for the long read. A second user-defined value, *F*, is the fraction of this median that is required for a *k*-mer to be considered solid. Thus, the final threshold distinguishing solid and weak *k*-mers in a given long read is defined as *t*=max(*T*,*F*∗*m*). In summary, with each pass over a long read, FMLRC dynamically calculates a threshold for determining weak or solid *k*-mers based on an absolute minimum and the surrounding *k*-mer frequencies from the long read.

For low-coverage short-read datasets, it is often the case that *t*=*T* because *F*∗*m*<*T*. For high-coverage short-read datasets, this dynamic threshold alleviates the need to select a fixed threshold beforehand, and it instead uses counts from the implicit de Bruijn graph to derive an expected count for *k*-mers in the read. Additionally, this approach enables FMLRC to adjust the threshold for different sizes of *k* automatically at run-time.

Finally, the branch limit, *L*, is scaled with each pass to allow for less branching when *k* is small and more branching when *K* is large. As described earlier, a small *k*-mer de Bruijn graph will have more branches and may require more computation to do a full depth-first traversal in repetitive regions. To avoid this, we restrict the short *k*-mer traversals to primarily fixes the “easy” errors caused by sequencing. As a result, the “harder” traversals caused by larger repetitive elements are addressed more accurately by the long *K*-mer pass. The branch limit factor, *B*, is a user defined parameter such that the maximum branch limit, *L*=*B*∗*k*.

### FMLRC parameter selection

FMLRC allows for five main parameters to be defined by the user: *T*, *F*, *B*, *k*, and *K*. *T* is the absolute minimum frequency required for a *k*-mer to be considered solid in the de Bruijn graph. *F* is the fraction of the median counts required for a *k*-mer to be considered solid in the de Bruijn graph. *B* is the branch limit factor that limits the amount of computation of a de Bruijn graph traversal. In all test cases, we used the FMLRC default parameters: *T*=5, *F*=0.10, and *B*=4.

The last two parameters are the choice of *k* and *K* for the short and long correction passes. To gain some insight into what values of *k* and *K* are best, we ran multiple tests using the *E. coli* K12 MG1655 and *S. cerevisiae* W303 datasets. We allowed *k*=[17,19,21,23,25] and *K*=[−,49,59,69,79,89], leading to a total of 30 test cases for each dataset. The test cases with *K*=− indicate that no second *K*-mer pass was performed (it is only using a one-pass, short *k*-mer for correction). For each test case, we ran FMLRC, aligned the corrected reads to the reference genome, and then gathered statistics on the resulting alignment. We counted the the total number of bases that matched the reference genome and the “gain” (see “Correction accuracy” section). The results of this experiment are shown in Table 1.

**Table 1:** Choosing *k* and *K*

	*K*					
*k*	–	49	59	69	79	89
*E.coli* - Matching Bases						
17	404736174	**405150086**	404809579	404608992	404361044	403888297
19	403117580	404580392	404571352	404418084	404297325	403826761
21	403002237	404365615	404367089	404255830	404131272	403841312
23	403516577	404381062	404378242	404240202	404107504	404041491
25	403819785	404461301	404480970	404453527	404363292	404236385
*E. coli* - Gain						
17	0.1011	0.388	0.4709	0.5258	0.5468	0.521
19	0.3823	0.5887	0.612	0.6245	0.6279	0.6172
21	0.4879	0.634	0.6429	0.6459	0.6442	0.6345
23	0.5137	0.641	0.6474	**0.6487**	0.6457	0.6361
25	0.523	0.6396	0.6453	0.6461	0.6422	0.6318
*S. cerevisiae* - Matching Bases						
17	1250679980	**1253590990**	1252340540	1251288299	1250445441	1249925285
19	1250052124	1252517259	1252462544	1252139063	1251853858	1251785285
21	1248322270	1251887685	1251963458	1251672602	1251758116	1251744201
23	1248801294	1252245368	1252387319	1252408890	1252545735	1252558864
25	1249574404	1252269051	1252478532	1252557840	1252778626	1252739127
*S. cerevisiae* - Gain						
17	0.0264	0.224	0.3159	0.3946	0.452	0.4871
19	0.1172	0.3903	0.443	0.4822	0.5096	0.5273
21	0.2527	0.4938	0.5129	0.527	0.5367	0.5434
23	0.3319	0.5153	0.5251	0.5332	0.5388	**0.5435**
25	0.3728	0.5155	0.5226	0.5287	0.5334	0.5372

This table shows the result of running FMLRC using many different values for *k* and *K* for an *E. coli* and *S. cerevisiae* datasets

The test cases with *K*=− indicate that no second pass of correction using the long *K*-mer was performed, so those test cases use a single pass short *k*-mer only. After correcting the reads, we aligned the results using BLASR [22] and gathered statistics on the alignments. Matching bases indicates the number of matching bases across all mappings. Gain is defined as (*TP*−*FP*)/(*TP*+*FN*) (see “Correction accuracy” section). For each statistic, the best result is bolded in the above table. To summarize, increasing values for *k* and *K* tend to increase the gain but decrease the total matching bases - a tradeoff between sensitivity and specificity. Additionally, all tested values of *K* for a long *K*-mer pass improves the results over a single *k*-mer pass

We see that as *k* and *K* increase, gain generally increases but the total number of matching bases decreases, indicating a tradeoff between sensitivity and specificity. In all of our tests, performing a second pass with the long *K*-mer always improved all three statistics. In general, the gain begins plateauing around *k*=21 and *K*=59 and matching bases decreases in the *E. coli* dataset, so we chose these as the default values for *k* and *K*. While it is clear that the “best” *k* and *K* is likely data-dependent because differences in coverage, sequencing quality, and sequencing content will impact the ability of FMLRC to find solid *k*-mers and perform corrections, these defaults perform close to optimal across all of our evaluated datasets.

## Results

We evaluated the accuracy of our method using complementary long- and short-read datasets for three species: *E. coli* K12, *S. cerevisiae* W303, and *A. thaliana* L*er*-0 (see “Availability of data and material” section). We compared the relative correction accuracy and computational performance of our method to several existing hybrid and long-read-only correction methods. We also assessed the effectiveness of our corrected reads for *de novo* assembly using a non-correcting assembler, Miniasm [6], and compared these data to several other state-of-the-art hybrid and long-read-only *de novo* assembly methods.

### Correction accuracy

To evaluate FMLRC, we used the approach used by the Error Correction Evaluation Toolkit (ECET) [23] to calculate error correction sensitivity, specificity, and “gain” relative to a known reference genome (*Sensitivity*=*TP*/(*TP*+*FN*), *Specificity*=*TN*/(*TN*+*FP*), and *gain*=(*TP*−*FP*)/(*TP*+*FN*) where TP, TN, FP, and FN are true positives, true negatives, false positive, and false negative, respectively). We modified the published pipeline to work efficiently with long reads, but the statistics are computed in an similar manner. In particular, we aligned the original and corrected FASTA files to the corresponding reference genome for each organism using BLASR [22]. Using the original ECET implementation, which was designed for short-read sequences, specific loci in long reads could not be evaluated before and after error correction due to the high incidence of short insertions and deletions. Instead, we consider loci relative to the reference sequence to which each read aligned. A nucleotide is considered “correct” if it aligns properly to a single nucleotide in the read sequence. Loci in the reference sequence with mismatched or delected nucleotides in the read sequence are considered incorrect. Our evaluation code is available at https://github.com/txje/lrc_eval, including the computation of error correction statistics directly from BLASR’s −*m*5 format alignments.

In addition to these statistics, we report the total aligned reads and properly aligned nucleotides. Again unlike short-read error correction, where every read is expected to align in full both before and after error correction, the number and span of long-read alignments may fluctuate and impacts the utility of a sequence dataset for downstream analysis. For example, error correction methods that agressively filter out low-quality sequences, such as Jabba [10], may report very high sensitivity and specificity, but do so by reporting and aligning only a subset of the input sequences.

In addition to evaluating FMLRC, we also evaluated the following hybrid correction methods using the same ECET pipeline: LoRDEC [4], Jabba [10], and CoLoRMap [11]. For completeness, we also included comparison to long-read-only methods: Canu [5], LoRMA [8], and Sprai [7]. For all tests, we ran LoRMA v0.4, LoRDEC v0.6 with options -k 21 -s 5, and Jabba with option -k 75 (as recommended in [10]). FMLRC was run with default parameters (-k 21 -K 59) for *E. coli*, *S. cerevisiae*, and *A. thaliana*. All other methods’ parameters were left at their defaults.

Table 2 shows accuracy metrics and resource usage for all compared methods. For *A. thaliana* and *S. cerevisiae*, FMLRC has the highest total corrected loci (true positives) and competitive gain and sensitivity. For *E. coli*, FMLRC corrects fewer loci than Jabba, but more total reads. As discussed above, methods with higher sensitivity and specificity - including LoRMA, Sprai, and Jabba - typically accomplish this by selectively reporting the highest-confidence corrected sequences. This kind of confidence filtering is possible after correction for most methods, but can negatively impact downstream assembly (see “*De novo* assembly” section).

**Table 2:** After aligning the corrected reads to a reference genome, sensitivity, specificity, and gain were computed

Method	Reads aligned	TP	FN	Sens.	Spec.	Gain	CPU (s)	Mem (GB)
E. coli K12								
Canu	58982	657793	1571579	0.2951	0.9987	0.1482	17421	2.99
CoLoRMap	81485	5538038	35474190	0.1350	0.9998	0.1332	137777	23.06
FMLRC	**81851**	13562639	15069242	0.4737	0.9996	0.4689	**11015**	4.60
Jabba	75620	**15553372**	**111609**	**0.9929**	**0.9999**	**0.9920**	22922	63.87
LoRDEC	81138	3278911	36691424	0.0820	0.9998	0.0808	61305	**2.08**
LoRMA	81051	1135657	144161	0.8874	**0.9999**	0.8669	54240	45.72
Sprai	75532	463636	293039	0.6127	**0.9999**	0.5783	44302	33.53
S. cerevisiae W303								
Canu	142765	1562542	5441239	0.2231	0.9992	0.1401	108175	**3.33**
CoLoRMap	210423	18901871	79065115	0.1929	0.9992	0.1857	2815200	45.49
FMLRC	211270	**31849332**	49204291	0.3929	0.9991	0.3829	**68519**	17.77
Jabba	**223385**	29893606	**42670**	**0.9986**	**0.9999**	**0.9967**	187968	367.92
LoRDEC	210151	8468872	96577493	0.0806	0.9997	0.0776	212495	3.56
LoRMA	204323	3063164	221583	0.9325	**0.9999**	0.9176	223358	49.52
Sprai	192670	2013269	3063751	0.3965	0.9996	0.3288	215261	49.52
A. thaliana Ler-0								
Canu	574065	12002535	72017120	0.1429	0.9986	0.1030	1301971	10.92
CoLoRMap	1075381	170235345	2056204621	0.0765	0.9983	0.0737	6802359	106.08
FMLRC	**1447042**	**442064624**	1164804073	0.2751	0.993	0.2601	**708910**	16.26
Jabba	813495	320742341	2945173	**0.9909**	0.9968	**0.9724**	3641309	333.44
LoRDEC	1113617	78276025	2022520259	0.0373	0.9979	0.0337	1111800	**3.42**
LoRMA	903298	2217661	**2778223**	0.4439	0.9986	0.3715	17281259	70.28
Sprai	751684	18960255	30734331	0.3815	**0.9996**	0.3631	5996657	8.11

For *A. thaliana* and *S. cerevisiae*, FMLRC produced more total true positive (corrected loci) than any other method while maintaining competitive sensitivity and gain. Methods with higher average specificity, notably Jabba, often discard a higher proportion of reads, reporting only those with the highest-confidence corrected sequence. FMLRC also requires significantly less CPU time than other hybrid error correction methods, and comparable memory. LoRDEC and FMLRC CPU time and memory results *include* construction of the BWT

### Performance

CPU and memory usage for each method are shown in Table 2. Performance tests were run on a homogenous cluster of 120 compute nodes, each with two Intel E2680 (2.5GHz) processors and 1Tb RAM. FMLRC requires less CPU time (including construction of the general-purpose BWT) than all other hybrid correction methods. On average, FMLRC’s memory usage is among the most memory-efficient methods, including Canu and LoRDEC. The remaining hybrid error-correction methods, CoLoRMap, LoRMA, and Jabba, use significantly more memory and, especially in the case of Jabba (> 300GB) may prove prohibitive to run without significant computational infrastructure. Jabba, in particular, while producing comparable total true positives to FMLRC, required 2−5× as much CPU time and 15−20× as much memory.

### *De novo* assembly

The ultimate goal of any long read correction algorithm is to provide better data for genomic analysis. We assessed the ability of our method to successfully complete assembly of simple and complex genomes and to compare its performance to other long-read error correction and *de novo* assembly methods. We assessed the methods listed in Table 3 on the *E. coli*, *S. cerevisiae*, and *A. thaliana* datasets listed above. Our method, along with LoRDEC and Sprai, perform only read correction. We used Miniasm (https://github.com/lh3/miniasm r159 and Minimap https://github.com/lh3/minimap r124) to assemble the corrected reads from these methods. We used option −*Sw*5 for Minimap; all other parameters were left at their defaults. The straightforward approach to identity-based overlapping and graph layout used by Miniasm allows us to assess the effect of read correction on *de novo* assembly.

**Table 3:** Long-read and hybrid correction and assembly methods

Method	Correction	Assembly	Preassembly	Citation
Miniasm		Long-read		[6]
Canu	Long-read	Long-read		[5]
Sprai	Long-read			[7]
LoRMA	Long-read			[8]
hybridSPAdes		Hybrid		[15]
DBG2OLC		Hybrid	X	[14]
Cerulean		Hybrid	X	[13]
ECTools	Hybrid		X	[9]
LoRDEC	Hybrid			[4]
Jabba	Hybrid			[10]
CoLoRMap	Hybrid			[11]
Nanocorr	Hybrid			[12]
FMLRC	Hybrid			Our method

All of the compared methods are shown along with their mode of error correction and assembly, each either long-read only or “hybrid” using complementary short-read data. “Preassembly” indicates whether a hybrid method requires the short read data to be preassembled using a different method

All assemblies were run on a heterogeneous Linux-based cluster with more than 9600 cores and 48Gb-1Tb RAM per node. All jobs had a hard limit of 16 processes and 7 days wall-clock run time. For larger genomes such as *A. thaliana*, several methods, including hybridSPAdes and Cerulean, failed after exceeding these limits or exceeding 1Tb main memory. Canu is a modern fork of the Celera Assembler and consists of the basic PBcR correction method using the MHAP overlapper followed by assembly with HGAP. So we assess only the Canu pipeline as a whole.

Several of the methods took prohibitively long (> 1 week) or failed to assemble the *A. thaliana* genome. We analyzed completed assemblies using Quast v4.1 [24] with default parameters in Table 4. Percent error indicates the total of mismatched bases, insertions/deletions, and no-calls (Ns). As shown, FMLRC has comparable performance to other methods for *E. coli K12*. It also outperforms all methods except Canu in terms of N50 for *S. cerevisiae W303*. Although the continuity is often higher for Canu and other long-read consensus methods, these typically rely on high coverage of long reads and degrade in performance as coverage drops. These test datasets contain high (> 100×) coverage of both long and short reads. Furthermore, post-assembly polishing steps such as Quiver [1] and Nanopolish [25] are typically effective in reducing the assembly error from less than 1% to less than 0.01%.

**Table 4:** Long-read and hybrid correction assembly statistics

Dataset	Method	# contigs	N50	Genome fraction	Error rate
*E. coli* K12	Canu + Miniasm	**1**	4631922	99.832	0.00444
Genome: 5Mb	CoLoRMap + Miniasm	**1**	4723063	84.485	0.02322
Pacbio: 450Mb	FMLRC + Miniasm	**1**	4646838	99.757	0.00029
Illumina: 3.4Gb	Jabba + Miniasm	76	72751	92.923	**0.00005**
	LoRDEC + Miniasm	**1**	4688727	97.504	0.00321
	LoRMA + Miniasm	107	64214	89.871	0.00099
	Sprai + Miniasm	**1**	4639974	99.989	0.00092
	Miniasm	**1**	**4783415**	0.002	0.01333
	Canu	2	**4656585**	**99.998**	0.00012
	hybridSPAdes	2	4469733	99.967	0.0001078
	DBG2OLC	2	4585967	98.210	0.00225
	Cerulean	16	1258842	98.959	0.09500
*S. cerevisiae* W303	Canu + Miniasm	36	729798	**89.739**	0.00521
Genome: 12Mb	CoLoRMap + Miniasm	**23**	766539	83.464	0.00761
Pacbio: 1.3Gb	FMLRC + Miniasm	32	771324	87.717	0.00175
Illumina: 18Gb	Jabba + Miniasm	186	62337	72.239	**0.00008**
	LoRDEC + Miniasm	61	597849	85.563	0.00941
	LoRMA + Miniasm	292	49850	74.632	0.00103
	Sprai + Miniasm	39	561985	88.697	0.00193
	Miniasm	29	566484	0.009	0.03738
	Canu	26	**777664**	**90.955**	0.00068
	hybridSPAdes	229	568823	87.303	0.00899
	DBG2OLC	32	530806	0.067	0.03407
	Cerulean	78	466556		0.03687
*A. thaliana* Ler-0	Canu + Miniasm	2100	74153	81.844	0.01410
Genome: 120Mb	CoLoRMap + Miniasm	963	404022	60.225	0.01853
Pacbio: 11Gb	FMLRC + Miniasm	1923	57751	67.275	0.00401
Illumina: 13Gb	Jabba + Miniasm	1632	57307	62.796	**0.00041**
	LoRDEC + Miniasm	2232	30229	43.088	0.01107
	LoRMA + Miniasm	**34**	26316	0.361	0.00448
	Sprai + Miniasm	1475	169744	91.070	0.00824
	Miniasm	740	615512	0.003	0.03409
	Canu	419	**835253**	**96.123**	0.00760
	DBG2OLC	440	754404	87.477	0.00388

Miniasm does not perform either read correction or consensus calling, so the resulting assembly has the same error profile of the input read

## Discussion

Correction of errors in long read sequences using complementary short reads remains a popular method for increasing the utility of long read sequence, particularly since long read sequencing remains prohibitively expensive relative to standard NGS in many cases. While several methods exist for hybrid error correction and assembly [4, 9–15], these approaches sometimes limit the utility of corrected sequences for downstream assembly or other applications due to low throughput - they report only segments where very high accuracy can be achieved or clip and trim low confidence sequences. These produce very polished (accuracy in excess of 99%) sequence, but reduce the total number and size of sequences available for assembly. In practice, a balanced approach is necessary to retain the long-range information while increasing sequence accuracy to aid in pairwise overlapping of reads.

Our proposed method does not perform any clipping or trimming of long read sequences, but corrects errors using high-accuracy short read sequences, enabling more sensitive and specific overlap of reads during *de novo* assembly. While no method produces obviously better results across all assembly metrics, FMLRC exhibits high accuracy correction while maintaining high assembly contiguity for a range of genome sizes. Practically, our method is also computationally efficient whereas competitive methods such as Jabba take prohibitive computational resources for even moderately sized data sets.

## Conclusion

Flexible “modular” approaches to *de novo* long read sequence assembly are becoming more popular with the introduction of efficient overlap and layout methods such as DALIGNER (https://github.com/thegenemyers/DALIGNER), MHAP [5], Minimap [6], and Miniasm [6]. Existing error correction methods including DBG2OLC [14], Cerulean [13], and hybridSPAdes [15] require preassembly of short read sequence and perform a variant of scaffolding using long read sequences. While this approach benefits from the high accuracy of short read sequence, it retains the biases inherent in assembly of short read sequences. In particular, it is often difficult or impossible to properly assemble low-complexity or repetitive sequences using only short reads [26].

To overcome these limitations, we developed FMLRC, a long read correction method that uses a multi-string BWT and FM-index to represent all de Bruijn graphs of a short read dataset. The method uses two passes to perform the correction: one with a relatively short *k*-mer and one with a longer *K*-mer. In each pass, unsupported sequences are identified in the long reads and the implicit de Bruijn graph identifies alternate, supported sequences from the short reads. These alternate sequences are then used to correct the original read.

We showed that FMLRC reliably corrects more loci than other methods while maintaining competitive gains, sensitivity, and specificity. Furthermore, FMLRC is more computationally efficient than any of the other hybrid error-correction methods evaluated. We further showed that using FMLRC as a preassembly error correction step in conjunction with existing overlap-layout assembly methods produces highly contiguous assemblies with competitive accuracy relative to existing hybrid and non-hybrid assembly methods.

Future work will include a specific cost-benefit analysis of the quantity of long- and short-read data required to effectively assemble genomes based on their size and repetitive structure. While previous work has been done in this area, FMLRC, as a more efficient method for hybrid correction of long reads, is expected to allow more effective *de novo* assembly with less long read data than previously possible. Future improvement and optimization of the FM-Index structure and bridging strategy could produce further speed and accuracy improvements over existing methods. In addition to a BWT with FM-index, it will be worth exploring the performance of other data structures, including novel variants of a de Bruijn graph that support multiple values of *k* [27, 28]. Our method is applicable to both Pacbio SMRT sequencing and nanopore sequencing datasets, however further parameter optimization may improve its accuracy and efficiency for nanopore sequences, which exhibit a slightly different error profile than Pacbio. In the long term, better integration of FMLRC error correction along with other tools for overlapping, layout, and consensus of long read sequencing data will help realize the goal of a fully modular and efficient *de novo* assembly process.

## Abbreviations

BWT: Burrows-wheeler transform
ECET: Error correction evaluation toolkit
FMLRC: FM-index long read corrector, Pacbio: Pacific biosciences

## Footnotes

1
http://github.com/holtjma/fmlrc

2
http://github.com/holtjma/msbwt

3
https://github.com/holtjma/msbwt/wiki/Converting-to-msbwt%27s-RLE-format
